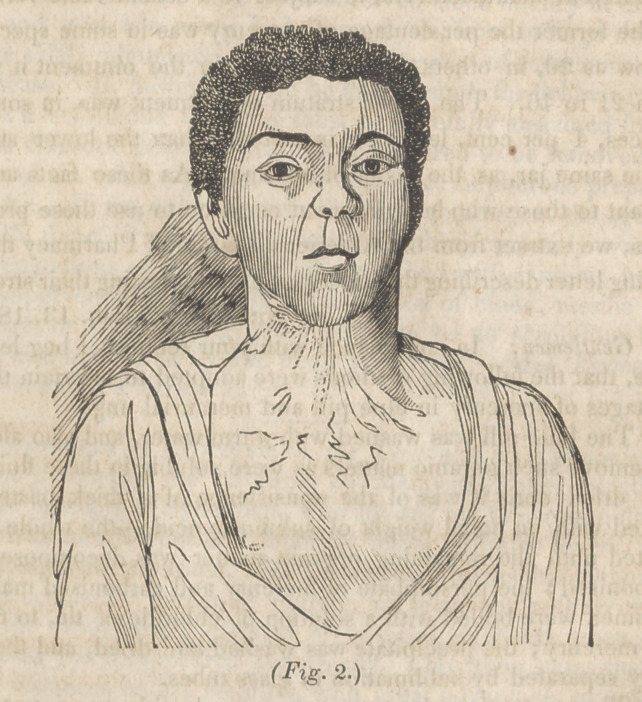# Case of Deformity from Burn Successfully Treated by Plastic Operation

**Published:** 1844-07

**Authors:** Daniel Brainard

**Affiliations:** Professor of Anatomy and Surgery in the Rush Medical College; Chicago


*Case of Deformity from Burn successfully treated by Plastic Operation.* By Daniel Brainard, M. D., Professor of Anatomy and Surgery in the Rush Medical College.

During a part of the winter of 1842, I performed, through the courtesy of Dr. J. V. Prather, the service of the surgical ward of the St. Louis City Hospital. On first entering it, I noticed a colored boy named William, aged about 12 years, affected with an extensive deformity, from a burn of four months standing, produced by the ignition of spirit gas. The chin was firmly bound to the superior extremity of the sternum, by a cicatrix an inch in length, by two and a half inches broad. This also extended, but superficially, on the sides of the jaw to its angles, and upon the breast, where there was a small part of it, imperfectly formed. The effect of this approximation of the sternum and lower jaw, was a permanent separation of the lips and jaws, except where the head was thrown much forward, inability to retain the saliva, or turn the head, great elevation of the sternum from continued efforts to throw the head backward. His condition is represented with sufficient accuracy by *fig.* 1. So great was the inconvenience, that he was found unfit for service and sent to the Hospital for relief.

*(Fig.* 1.)

On Monday, November 7, 1842, I proceeded, in presence of the medical class of the St. Louis University, and several medical gentlemen, with the assistance of Drs. Prather and Pope, to put in operation Dr. Mütter's operation for the relief of such deformities. An incision was accordingly made transversely below the chin, and in contact with the sound skin, from side to side, which, on raising the lower jaw, left a deep fissure between it and the sternum. A flap three and a half inches broad, by seven inches in length was then dissected up from the right shoulder, and turned upon its base in such a manner as to fill up the vacancy beneath the chin. It was fixed in this position by twisted sutures, supported by adhesive straps, and a roller. The denuded surface upon the shoulder was covered with lint and cerate, and the patient placed in bed. The flap adhered, by the first intention, excepting one and a half inches at its extremity, which, from its great distance from the pedicle, sloughed and separated. Notwithstanding this unfavorable occurrence, the result of the operation was highly satisfactory.

May 7, 1844, eighteen months afterward, the chin was three inches distant from the sternum, the natural depression between the parts existed, the mouth was closed, and the patient in the condition represented in *fig.* 2. There existed, however, upon the left side, a band which prevented the movements of the head and jaw from being as free as natural. In a case operated upon by Professor March of Albany, which he had the kindness to show me, a similar band existed. These facts lead me to believe that it would be well in such cases to take two flaps, one from each shoulder, as suggested by Dr. Mütter.—(*American Journal Méd. Sciences*, *July* 1842, *p.* 80.)

(*Fig.* 2.)

The above described operation was first proposed and performed by Dr. Mütter, (op. cit.) but had not, as far as I am aware, been repeated by any one at the time of its performance in the above case. This has since been done with success, by Dr. March, by a surgeon of Connecticut, whose case I have mislaid, and more recently, in a modified form, by Dr. Wm. B. Diver of Cincinnati. Of its value, no one who has had occasion to observe its results, can entertain a doubt, as it offers a remedy for a frightful and otherwise incurable deformity. It is probable, however, that great care on the part of the surgeon, during the period of cicatrization of such burns, might, in many cases, prevent the necessity of resorting to it.

Chicago, June 29, 1844.

## Figures and Tables

**Fig. 1. f1:**
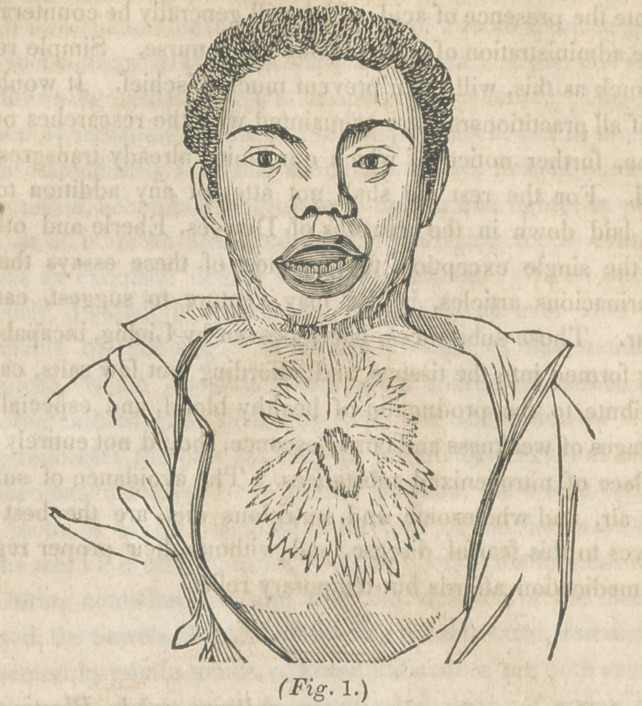


**Fig. 2. f2:**